# Anthocyanins in Non-Small Cell Lung Cancer (NSCLC) Treatment and Prevention

**DOI:** 10.3390/nu16101458

**Published:** 2024-05-12

**Authors:** Kostas A. Papavassiliou, Amalia A. Sofianidi, Athanasios G. Papavassiliou

**Affiliations:** 1 First University Department of Respiratory Medicine, ‘Sotiria’ Hospital, Medical School, National and Kapodistrian University of Athens, 11527 Athens, Greece; konpapav@med.uoa.gr; 2 Department of Biological Chemistry, Medical School, National and Kapodistrian University of Athens, 11527 Athens, Greece; amsof.00@gmail.com

An ever-growing volume of data supports the important role of dietary interventions in cancer prevention and the beneficial effects of plant secondary metabolites in solid tumor therapeutics. To this end, anthocyanins, a group of water-soluble colored flavonoids present in all tissues of higher plants (i.e., roots, leaves, stems, flowers, and fruits) [1], have garnered considerable attention due to their chemopreventive and chemotherapeutic bioactivity toward various types of cancers, including non-small-cell lung cancer (NSCLC), the most common type of primary lung cancer [2]. Anthocyanins, occurring as glycosides of their respective aglycones, called anthocyanidins (typically cyanidin, pelargonidin, or delphinidin), are chiefly found in red and purple berries, purple sweet potatoes, cherries, apples, grapes, plums, black soybeans, cabbage, or foods containing high levels of natural colorants. This class of natural substance possess strong anti-oxidative potential and are implicated in anti-inflammatory, immunomodulatory, antiproliferative, apoptotic, and antitumorigenic processes [2].

Extensive research has been conducted on the utilization of anthocyanins for the treatment of lung cancer. Pal et al. showcased the potency of delphinidin, an anthocyanidin found in colorful fruits and vegetables, as a robust inhibitor of the epidermal growth factor (EGF) receptor (EGFR) and the vascular endothelial growth factor (VEGF) receptor (VEGFR) in NSCLC cells with heightened EGFR/VEGFR expression. Delphinidin was also shown to diminish the expression of hypoxia-inducible factor 1a (HIF-1a), a pivotal regulator of *VEGF* transcription, limiting cancer angiogenesis even further [3]. Kausar et al. explored the synergistic effect of berry anthocyanin mixtures in NSCLC. The stimulation of cell-cycle arrest, apoptosis, and the hampering of NSCLC cell invasion and migration were facilitated by the oncogenic Notch and Wnt signal transduction pathways along with their downstream targets, including β-catenin, c-myc, cyclin D1, cyclin B1, phospho-extracellular signal-regulated kinase (ERK), matrix metalloproteinase-9 (MMP-9), and VEGF proteins. Furthermore, amplified cleavage of the anti-apoptotic mediators B-cell lymphoma 2 (Bcl2) and poly(ADP-ribose) polymerase (PARP) was observed, which leeds to their inactivation, coupled with the intensified inhibition of tumor necrosis factor alpha (TNFα)-induced nuclear factor kappa B (NF-κB) activation, impeding the metastatic and angiogenic properties of cancer cells [4].

Cyanidin-3-glucoside (C3G), one of the most abundant anthocyanins in a wide variety of vegetables and fruits, has been shown to hinder the proliferation, migration, and invasion, and foster the apoptosis of lung adenocarcinoma cells by downregulating the expression of tumor protein p53-inducible protein 3 (TP53I3). In addition, C3G could inhibit the activation of the phosphoinositide 3-kinase (PI3K)/protein kinase B (AKT)/mechanistic target of rapamycin (mTOR) oncogenic signaling pathway in vitro through the downregulation of TP53I3 [5]. Wu et al. demonstrated in vivo that when C3G is combined with the chemotherapy agent 5-fluorouracil (5-FU), it exerts synergistic anticancer effects on lung large-cell carcinoma, a subtype of NSCLC. C3G alone or in combination with 5-FU was shown to alter the expression of the tumor microenvironment (TME)-related factors antigen Kiel 67 (Ki67), leucocyte common antigen (CD45), programmed death-ligand 1 (PD-L1), and 5′-ecto-nucleotidase (CD73) [6], paving the way for future synergistic combinations of these molecules with immunotherapy.

Large population studies revealed that anthocyanins could also prevent lung cancer. In the American population, a beneficial link between dietary anthocyanins and decreased lung cancer risk has been noted, possibly through the mitigation of oxidative stress [7]. Nevertheless, additional research is required to substantiate this direct correlation and explore the exact underlying mechanisms.

Considering the prevalence of anthocyanins in dietary sources, it is approximated that individuals typically intake around 180 mg per day of these antioxidants [8]. However, relying solely on dietary intake may not offer the most efficient means of delivering anthocyanins to different tissues [9]. Consequently, diverse strategies and nano formulations have emerged to enhance the bioavailability and anticancer effectiveness of anthocyanins. Nanoparticles have been proposed as a delivery mechanism for anthocyanins that provides protection to the enclosed compound. Carboxymethyl chitosan (CMC) nanoparticles have been used to encapsulate C3G and were explored in lung cancer cell lines. The nanoparticles displayed no cytotoxicity and effectively enhanced the antioxidant competence of anthocyanins, representing a promising avenue for delivering therapeutic anthocyanins to lung cancer tissue [10]. Exosomes, natural nanoparticles, were also investigated to determine their capacity to efficiently encapsulate and transport anthocyanins in vitro. Exosomes loaded with anthocyanidin-rich extract exhibited significantly augmented efficacy in inhibiting cell proliferation across various cancer cell lines, including breast, prostate, lung, ovarian, pancreatic, and colon cancer cells, compared to the extract alone [11]. In this context, exosomes could serve as potent delivery mechanisms for anthocyanins in tissues alongside synthetic nanoparticles.

To summarize, anthocyanins, abundant in our daily diets, exhibit potent antioxidant properties and could play a crucial role in combating tumor development. Notably, these compounds did not show or showed minimal negative effects on healthy cellular models. With their widespread availability, they hold the potential to “democratize” anticancer treatments, ensuring access across all socioeconomic strata and narrowing the gap in lung cancer prevention and treatment. Although there is extensive evidence from both in vitro and in vivo studies regarding the anticancer properties of anthocyanins, most of these studies were conducted on animals; therefore, the aim for the near future is to perform more clinical studies to confirm the positive impact of anthocyanins on curbing lung carcinogenesis.

## References

[1] Clifford M.N. (2000). Anthocyanins–Nature, Occurrence and Dietary Burden. J. Sci. Food Agric..

[2] Dharmawansa K.S. (2020). Dietary Anthocyanins- Smart Molecules with Chemo Preventive and Chemotherapeutic Activity. Org. Med. Chem. Int. J..

[3] Pal H.C., Sharma S., Strickland L.R., Agarwal J., Athar M., Elmets C.A., Afaq F. (2013). Delphinidin Reduces Cell Proliferation and Induces Apoptosis of Non-Small-Cell Lung Cancer Cells by Targeting EGFR/VEGFR2 Signaling Pathways. PLoS ONE.

[4] Kausar H., Jeyabalan J., Aqil F., Chabba D., Sidana J., Singh I.P., Gupta R.C. (2012). Berry Anthocyanidins Synergistically Suppress Growth and Invasive Potential of Human Non-Small-Cell Lung Cancer Cells. Cancer Lett..

[5] Chen X., Zhang W., Xu X. (2021). Cyanidin-3-Glucoside Suppresses the Progression of Lung Adenocarcinoma by Downregulating TP53I3 and Inhibiting PI3K/AKT/mTOR Pathway. World J. Surg. Oncol..

[6] Wu C.-F., Wu C.-Y., Lin C.-F., Liu Y.-W., Lin T.-C., Liao H.-J., Chang G.-R. (2022). The Anticancer Effects of Cyanidin 3-O-Glucoside Combined with 5-Fluorouracil on Lung Large-Cell Carcinoma in Nude Mice. Biomed. Pharmacother..

[7] Zhang Y., Zhu M., Wan H., Chen L., Luo F. (2022). Association between Dietary Anthocyanidins and Risk of Lung Cancer. Nutrients.

[8] Galvano F., La Fauci L., Lazzarino G., Fogliano V., Ritieni A., Ciappellano S., Battistini N.C., Tavazzi B., Galvano G. (2004). Cyanidins: Metabolism and Biological Properties. J. Nutr. Biochem..

[9] Posadino A.M., Giordo R., Ramli I., Zayed H., Nasrallah G.K., Wehbe Z., Eid A.H., Gürer E.S., Kennedy J.F., Aldahish A.A. (2023). An Updated Overview of Cyanidins for Chemoprevention and Cancer Therapy. Biomed. Pharmacother..

[10] Amararathna M., Hoskin D.W., Rupasinghe H.P.V. (2022). Anthocyanin Encapsulated Nanoparticles as a Pulmonary Delivery System. Oxid. Med. Cell. Longev..

[11] Munagala R., Aqil F., Jeyabalan J., Agrawal A.K., Mudd A.M., Kyakulaga A.H., Singh I.P., Vadhanam M.V., Gupta R.C. (2017). Exosomal Formulation of Anthocyanidins against Multiple Cancer Types. Cancer Lett..

